# Emotional Regulation and Academic Performance in the Academic Context: The Mediating Role of Self-Efficacy in Secondary Education Students

**DOI:** 10.3390/ijerph18115715

**Published:** 2021-05-26

**Authors:** Pablo Usán Supervía, Alberto Quílez Robres

**Affiliations:** 1 Department of Psychology and Sociology, Faculty of Human Sciences and Education, University of Zaragoza, 22003 Huesca, Spain; pusan@unizar.es; 2 Department of Science Education, Faculty of Human Sciences and Education, University of Zaragoza, 22003 Huesca, Spain

**Keywords:** emotional regulation, self-efficacy, academic performance, students, adolescent

## Abstract

**Background:** in the school stage, adolescents experience different emotional and motivational states involved in the learning process that play a fundamental role in their personal and academic development. In this way, the study focuses on analyzing the relationships between emotional regulation, self-efficacy and academic performance, as well as the possible mediating role of self-efficacy in both. **Methods:** the study included 2204 students, both male (N = 1193; 54.12%) and female (N = 1011; 45.87%) with ages ranging from 12 to 18 years (M = 14.69; DT = 1.76). The measures used for the investigation were the Emotion Regulation Questionnaire (ERQ), the Academic Self-Efficacy Scale (ASES) and average marks were used to measure students’ academic performance. **Results:** The results of the study revealed a self-determined behavioral pattern characterized by high scores in emotional regulation, self-efficacy and academic performance. Likewise, the mediating role of self-efficacy between emotional regulation and student academic performance was significant. **Conclusion:** the influence of the academic self-efficacy variable as a mediator between the studied constructs is denoted, as well as the importance of promoting adaptive behaviors in the classroom that can lead to adequate personal development of students together with optimal academic performance.

## 1. Introduction

Currently, research in education is undergoing constant changes. Students are exposed to a wide variety of personal, academic and social variables, set in multiple contexts [1].

In this way, understanding the operation of psychological variables in academic contexts is a crucial step not only to better comprehend cognitive processes among students, but also to shape the qualities that will contribute to configure their adult personality [2]. Adolescence is a critical period in the life-cycle, in which social, personal, emotional and motivational experiences, and the way they are confronted, play a key role [3].

While most students go through this stage without suffering major personal or academic issues, others may undergo more or less prolonged feelings of lack of motivation, stress, anxiety, pessimism and other variables that can undermine their commitment to their school tasks [4].

### 1.1. Emotional Regulation

Emotional regulation is understood as a dynamic process that involves different strategies oriented to increase, appease or sustain an emotion [5]. As such, emotional regulation refers to the internal and external processes through which the individual evaluates his emotions in a given setting, including both conscious and unconscious physiological, behavioral and cognitive dimensions, and their projection onto the affective, cognitive and social spheres [6].

Emotions play a key role in academic settings, and have a significant impact on academic performance. As such, the correct regulation and handling of emotions is a central variable for the individual’s personal and academic wellbeing [7]. 

In this way, emotions operate as a control mechanism that alert individuals about the importance of a given setting, steering his or her reaction to adapt to it [8].

In recent years, interest in emotional regulation have transcended the limits of academic interest, and has percolated through wider sectors of society. New research avenues focus on adolescent populations and on the role played by different psychological variables, such as the mechanisms used to moderate stress and anxiety in academic settings [9]; self.-regulation skills [10]; coping strategies [11]; peer-support and socialization [12] and, more broadly, school pupils’ psychological wellbeing [13,14].

Finally, it must be pointed out that emotion regulation processes can facilitate or prevent both physical and psychological problems, and that their role is particularly relevant during adolescence, when the adult personality is being formed [15].

### 1.2. Self-Efficacy

Another key variable for learning processes and school performance is self-efficacy which refers to an individual beliefs in his or her capacity to execute behaviors necessary to produce specific performance attainments [16]. In an academic context, self-efficacy is a self-regulatory mechanism that has a major impact on the pupils’ academic behavior, as it determines the subject’s perception of his or her own ability to learn and carry out a given task, a key variable for the student’s ability to adapt to future situations [17].

Students with high levels of self-efficacy perceive school tasks as a challenge that they face confidently, armed with their knowledge and skills, resulting in a more responsible and efficient attitude towards school tasks [18]. Empirical research shows that academic self.-efficacy can be used to predict variables such as interest for learning, engagement, commitment, perseverance and motivation [19]; perseverance and motivation [20] and, more broadly, academic satisfaction and wellbeing [21]. 

The relationship between self-efficacy and academic performance is usually positive if we take a look at the scientific literature. In this way, certain correlation studies advocate the close relationship between these variables with the adolescent population of Secondary Education [22]. Manzano, Outley, Gonzalez & Matarrita [23] found significant correlations in students of different ages including primary school and Castro [24] found statistically significant correlations between self-efficacy and academic performance adolescent students.

From a different perspective, low self-efficacy is seen as related to non-adaptive academic behaviors, leading to less commitment to school tasks and poor academic performance [25] and even to psychological problems in adolescents, such as anxiety, stress and even depression [26]. 

### 1.3. Academic Performance

Finally, academic performance assesses the students’ success in the teaching-learning process [27]. 

Scientific literature has largely relied on two values to measure academic performance: one, more quantitative and objective referred to qualify the academic performance by numbers using different school marks, and two, more qualitative and subjective, takes into account the personal circumstances of the student and its immediate social environment [28]. 

Performance is a multidimensional concept which depends on targets and expected results [29]. School marks are particularly reliable predictors of school performance [30,31] but alternative variables have also been considered, such as the number of failed subjects, the number of repeated courses, and even the time spent in assimilating subject matters [32,33,34].

The study of such a broad construct as academic performance has been addressed from different perspectives. Fierro, Almagro & Sáenz-López [35] emphasize emotional factors, especially motivational processes and emotional intelligence; Portolés & Gónzalez [36] focus on the personal factors that determine the personality of students; Guerra & Guevara [37] take into account complementary variables, such as learning styles, parental styles and study strategy; while Pulido & Herrera [38] underscore the influence value of socio-demographic variables.

For all of this, and following Méndez [39], more studies are necessary to increase our understanding of the different variables involved in academic performance, and to develop strategies to improve the students’ personal development and academic satisfaction, ultimately contributing to reduce early school dropout [40]. 

### 1.4. Objective and Hypothesis

In this context, and given the absence of studies that directly relate the variables under consideration, the main aim of this empirical study is to analyze the relationship between emotional regulation, self-efficacy and academic performance in a sample of secondary school students. The study’s hypotheses are:

(a) Self-efficacy is related to emotional regulation and academic performance;
(b) There is a behavioral pattern characterized by high levels of self-efficacy, emotional regulation and academic performance, in a line of adaptive behavior;
(c) Self-efficacy plays a mediating role in the relationship between emotional regulation and academic performance.

## 2. Method

### 2.1. Sample

The study sample consisted of 2204 male (N = 1193; 54.12%) and female (N = 1011; 45.87%) students belonging to 15 public secondary high school with ages between 12 and 19 years (M = 14.69; SD = 1.76). The educational centers were chosen by a simple random sample from among all those available in the city. Inclusion criteria were the ability to read in perfect Spanish to make sure that they could understand the questionnaire. Incomplete questionnaires were discarded and students with cognitive disorders who could not fully understand the questionnaire were excluded.

### 2.2. Instruments

Firstly, emotional regulation was measured using Emotion Regulation Questionnaire (ERQ) [41] adapted to Spanish adolescents by [42]. This mono-factorial scale comprises 10 items that express the degree of agreement or disagreement in reference to how the adolescent regulates its emotions in two main dimensions; cognitive reevaluation (six items) (e.g., “I control my emotions by changing the way I think about the situation I am in”) and emotional suppression (four items) (e.g., “I keep my emotions to myself”). Answers are expressed in a five-point Likert scale ranging from “Strongly disagree” (1) to “Strongly agree” (5). In terms of reliability, the translated version of the questionnaire yielded a Cronbach-α value of 0.84, and of 0.82 in our study.

Secondly, in order to measure self-efficacy, the Academic Self-Efficacy Scale (ASES), validated for adolescents by García, Inglés, Torregrosa, Ruiz, Díaz, Pérez & Martínez [18]. The scale comprises 10 items to measure self-efficacy in an academic setting (e.g., “I am convinced that I can carry out outstanding exams”). The responses measure the degree of agreement and disagreement of the subject in a five-point Likert scale ranging from “Strongly disagree” (1) to “Strongly agree” (5). Several studies have shown the reliability of the questionnaire in academic settings, yielding an overall Cronbach-α value of 0.91, and of 0.90 in our study.

Finally, to determine the academic performance of adolescent students, the global average grade reflected in the report card for the first school trimester was taken on a scale of 0 points (minimum) to 10 points (maximum), resulting in one of the most common procedures used and greater predictor of stability of student academic performance [30,31]. The reliability of the academic performance variable denoted a Cronbach-α value alpha in our research of 0.84.

### 2.3. Procedure

The research was approved by the different educational centers as well as the parents/guardians of the students through informed consent. All students and their parents/guardians were previously informed of the nature of the study participating voluntarily and thus respecting the ethical guidelines of the Declaration of Helsinki [43] in all its terms. The study protocol was approved by CEIC Aragón - No 04/2019.

### 2.4. Data Analysis

Descriptive statistics were carried out to establish the socio-demographic profile of the sample, including variables as gender, age, course, type of school and course repeats. Subsequently, correlations were carried out between the variables of emotional regulation, self-efficacy and academic performance, analyzed and processed using the statistical program IBM SPSS v26.0. In turn, a K-means cluster analysis was carried out to distribute the students in the sample into three statistically significant groups among themselves according to their standardized values, allowing groups to be created based on the similarity between the variables studied. Finally, a mediation analysis was proposed through the MACRO of SPSS v26.0 to verify the indirect effect of the self-efficacy variable in the relationship between emotional regulation and academic performance, carrying out a bootstrapping procedure with 10,000 repetitions. For all operations, a level of significance *p* < 0.05 with a confidence level of 95% was considerated.

## 3. Results

### 3.1. Demographic Variables

The sample comprised 2204 students both male (N = 1193; 54.12%) and female (N = 1011; 45.87%) with ages ranging from 12 to 19 years (M = 14.69; DT = 1.76) (Table 1).

### 3.2. Descriptive Variables

As illustrated in Table 2, results for the emotional regulation, self-efficacy and academic performance were highly variable.

Cohen’s *d* suggested that gender played no significant role in the three variables under study, although females yielded slightly higher scores in emotional regulation and academic performance, while males scored slightly higher in self-efficacy.

### 3.3. Correlational Analysis between Emotional Regulation, Self-Efficacy and Academic Performance

Table 3 illustrates correlations between the variables under consideration. They were all significant correlations, but in different ways. Emotional regulation was correlated with self-efficacy (*r* = 0.472) and academic performance (*r* = 0.201) while academic performance was found to be correlated with self-efficacy (0.280).

### 3.4. Cluster Analysis in Significant Groups of Emotional Regulation, Self-Efficacy and Academic Performance Variables

K-means cluster analysis (Table 4) was undertaken in order to divide the sample into three statistically significant groups. Group 1 (N = 468, 21.32%) was characterized by low scores in emotional regulation and self-efficacy, leading to poor academic performance; Group 2 (N = 1088, 49.36%) yielded near-average scores in all three variables; and finally Group 3 (N = 648, 29.40%) yielded significantly above average scores in all three variables.

### 3.5. Mediation Effects of Self-Efficacy in the Relationship between Emotional Regulation and Academic Performance

In order to establish whether the relationship between emotional regulation and academic performance was mediated by self-efficacy, Hayes’s MACRO [44] tool in Process 3.0 de SPSS (v 26.0) was used, following the methodology put forth by Tal-Or, Cohen, Tsarfati & Gunther [45]. 

**Figure 1:**
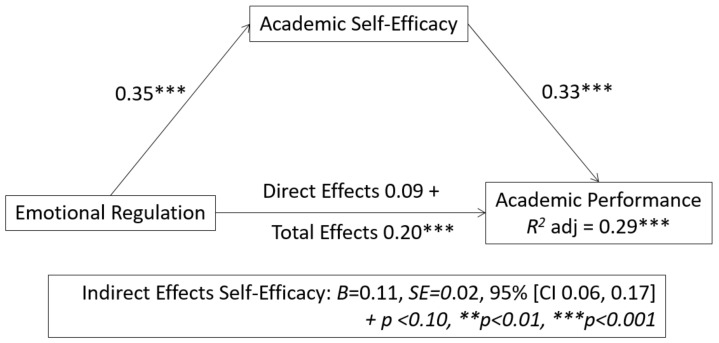
Mediating role of self-efficacy in the relationship between emotional regulation and academic performance.

As shown in Figure 1, self-efficacy mediated in the relationship between emotional regulation and academic performance. The results indicated a mediating effect of emotional regulation (VI) on self-efficacy of 0.35 ***, and self-efficacy on academic performance (VD) of 0.33 ***, in both cases *p* > 0.001. Zero was not included in the bootstrap interval, *B* = 0.20, *SE* = 0.02, 95% [CI 0.06, 0.17] so it could be argued that self-efficacy mediated in the relationship between emotional regulation and academic performance. These results suggested that in the first moment emotional regulation had no direct significant effect on academic performance (0.05, *p* < 0.10), but its combination with self-efficacy yielded a result of 0.20, *p* < 0.001 (direct effect + indirect effect), the proportion of variance being explained by model *R*^2^ = 0.29 ***. This suggested that self-efficacy played a mediating role in the relationship between emotional regulation and academic performance having important practical implications in the academic context.

## 4. Discussion

The aim of this study was to analyze the relationship between emotional regulation, self-efficacy and academic performance in adolescent secondary school students.

The first hypothesis, that self-efficacy is correlated to emotional regulation and academic performance, was fully confirmed; the results show that self-efficacy is positively correlated with emotional regulation and academic performance.

This conclusion agrees with the existing literature. Ocaña [46] establishes a strong correlation between self-efficacy and emotional regulation in students, and argues that high scores in these variables help to prevent academic stress; Gómez-Tabares & Narváez [47] allude to the intrinsic relationship between academic self-efficacy and emotional regulation, and of these variables with empathy in adolescent students; Domínguez [48] outlines cognitive emotional regulation strategies, including self-efficacy, as factors that contribute to protect students from emotional and academic exhaustion. In general, there is wide agreement about the significant relationship between emotional regulation and self-efficacy [49,50,51,52].

There is also wide agreement concerning the correlation between academic self-efficacy and academic performance. Manzano, Outley, Gonzalez & Matarrita [23] have pointed out significant correlations in students in different age groups; Castro [24] and Galleguillos & Olmedo [53] found statistically significant correlations between self-efficacy and academic performance in secondary school students.

The second hypothesis, which maintains the existence of an adaptive behavioral pattern characterized by high scores in all three variables under consideration was also confirmed. Our results indicate that the three variables are significantly correlated, and the cluster analysis identified a group of students that respond to this profile, yielding above average scores in all three variables. It is worth pointing out that no previous study has explicitly analyzed the mutual relationships between these three variables. However, some studies examine these constructs from different perspectives: Hayat, Shateri, Amini & Shokrpour [54] develop an equation model in which self-efficacy, learned emotional strategies and academic performance converge in a behavioral pattern characterized by the use of metacognitive learning strategies; Putwain, Sander & Larkin [55] argue that the variables under consideration in this study have an effect on study-related skills and behavior; finally, Schnell, Ringeisen, Raufelder & Rohrmann [56] analyze the impact of self-efficacy and emotional self-regulation in meeting academic performance targets.

The third hypothesis, which establishes that self-efficacy plays a mediating role in the relationship between emotional regulation and academic performance was also confirmed, as self-efficacy was found to have an effect on the other two variables.

These results must be examined in detail. On the one hand, the variables under study present bidirectional correlations with one another. On the other hand, the results of mediation analysis suggest that emotional regulation is a poor predictor of academic performance; that is, the effect of the former over the later is not statistically significant.

At any rate, self-efficacy was found to play a significant mediating role in the relationship between the other two constructs. These results emphasize the importance of self-efficacy for adolescent students, specifically in the relationship between emotional regulation and academic performance, which has direct practical implications.

To our knowledge, no previous studies have directly addressed the mediating role of self-efficacy in the relationship between the other two variables. However, many studies examine these constructs from different perspectives: Alhadabi & Karpinski [57] establish the mediating role played by self-efficacy in the relationship between academic performance and intrinsic academic motivations; Avalos, Oropeza, Ramírez & Palos [58] establish the mediating role of self-efficacy in the relationship between academic skills and achievements. Luberto, Cotton, McLeish, Mingione & Bryan [59] allude to the mediating role played by self-efficacy between emotional regulation and mindfulness techniques; Udavar, Fiori & Bausseron [60] underscore the modulating role of self-efficacy in the relationship between emotional intelligence and academic performance in secondary school students; finally, Alipio [61] establishes the influence of self-efficacy on expectancy-value beliefs concerning academic results.

## 5. Conclusions

All results emphasize the important role played by the constructs under consideration which, alongside personal and contextual circumstances, have a direct effect on school performance. It is, therefore, imperative to address these issues in order to create the conditions for the adequate personal and academic development of the students.

Our results have practical implications for educational policies, namely the promotion of teaching strategies to promote self-efficacy, self-esteem and resilience, and encourage self-determined and motivated behaviors from an early age, such as the development of self-efficacy, dedication and intrinsic motivation towards school tasks, helping students to feel more confident at school.

Future studies may examine the variables of our study with other sociodemographic variables such as age, gender, race or people of a certain ethnicity to examinate their relationships.

## Figures and Tables

**Table 1:** Socio-demographic characteristics of the sample.

**Gender**		**N**	**%**
	Male	1193	54.12
	Female	1011	45.87
**Age**	12 years	259	11.75
	13 years	325	14.74
	14 years	335	15.19
	15 years	478	21.68
	16 years	419	19.01
	17 years	206	9.34
	18 years	118	5.35
	19 years	64	2.90
**Academic year**	1° ESO	316	14.33
	2° ESO	392	17.78
	3° ESO	343	15.56
	4° ESO	594	26.95
	1° BACH	411	18.64
	2° BACH	148	6.71
**Repeating course?**	Yes	429	19.46
	No	1775	80.53
**Type of school**	Public	1439	65.29
	Private	648	34.70

**Table 2:** Results of descriptive variables emotional regulation, self-efficacy and academic performance.

	Total		Male		Female		
	**x**	**sd**	**x**	**sd**	**x**	**sd**	**Cohen’s d**
**Emotional regulation**	2.74	1.00	2.72	0.97	2.77	1.03	−0.049
**Academic Self-Efficacy**	3.54	0.75	3.55	0.71	3.54	0.79	0.013
**Academic Performance**	2.95	1.04	2.91	1.11	3.00	0.98	−0.085

**Table 3:** Correlational analysis between emotional regulation, self-efficacy and academic performance.

	1	2	3
**Emotional regulation**	1		
**Academic Self-Efficacy**	0.472 **	1	
**Academic Performance**	0.201 **	0.280 **	1
*Mean (X)*	2.74	3.54	2.95
*SD*	1.00	0.75	1.04
*Cronbach’s alpha*	0.82	0.90	0.84

** The correlation is significant at 0.01 (bilateral).

**Table 4:** Cluster analysis between emotional regulation, self-efficacy and academic performance.

	Group 1 (N = 468, 21.32%)		Group 2 (N = 1088, 49.36%)		Group 3 (N = 648, 29.40%)		Total Sample			
	**x**	**sd**	**x**	**sd**	**x**	**sd**	**x**	**sd**	**F**	**Sig**
**Emotional regulation**	1.62	0.63	2.61	0.69	3.78	0.55	2.74	1.00	327.07	0.000
**Academic Self-Efficacy**	2.61	0.54	3.56	0.53	4.20	0.44	3.54	0.75	194.86	0.000
**Academic Performance**	2.09	0.86	2.98	1.00	3.53	0.79	2.95	1.04	257.74	0.000
